# Cost-effectiveness of precision screening for esophageal cancer based on individualized risk stratification in China: Real-world evidence from the ESECC trial

**DOI:** 10.3389/fonc.2022.1002693

**Published:** 2022-11-30

**Authors:** Fuxiao Li, Mengfei Liu, Chuanhai Guo, Ruiping Xu, Fenglei Li, Zhen Liu, Yaqi Pan, Fangfang Liu, Ying Liu, Hong Cai, Zhonghu He, Yang Ke

**Affiliations:** ^1^ Key Laboratory of Carcinogenesis and Translational Research (Ministry of Education/Beijing), Laboratory of Genetics, Peking University Cancer Hospital & Institute, Beijing, China; ^2^ Shenzhen Institute of Advanced Technology, Chinese Academy of Sciences, Shenzhen, Guangdong, China; ^3^ Anyang Cancer Hospital, Anyang, Henan, China; ^4^ Hua County People’s Hospital, Anyang, Henan, China

**Keywords:** precision screening, esophageal cancer, cost-effectiveness, ESECC trial, risk prediction

## Abstract

**Background:**

Conventional universal endoscopic screening with pathology-based endoscopic re-examination for esophageal squamous cell carcinoma is in need of reform in China. We established a “two-step” precision screening strategy using two risk prediction models and have evaluated the cost-effectiveness of this precision strategy compared with the traditional strategy based on a large population-level randomized controlled trial from a healthcare provider’s perspective.

**Methods:**

Four precision screening strategies with different risk cutoffs at baseline screening and endoscopic surveillance were constructed, and then compared with traditional strategy through modeling using subjects from the screening cohort of the ESECC (Endoscopic Screening for Esophageal Cancer in China) trial. Total screening costs and the number of SDA (severe dysplasia and above in lesions of the esophagus) cases were obtained to calculate the average screening cost per SDA detected, the incremental cost-effectiveness ratio (ICER) and protection rates. Sensitivity analysis was conducted to evaluate uncertainties.

**Results:**

Compared to traditional strategy, all precision screening strategies have much lower average costs for detection of one SDA case ($7,148~$11,537 *vs.* $14,944). In addition, precision strategies 1&2 (strategies 1,2,3,4 described below) achieved higher effectiveness (143~150 *vs.* 136) and higher protection rates (87.7%~92.0% *vs.* 83.4%) at lower cost ($1,649,727~$1,672,221 *vs.* $2,032,386), generating negative ICERs (-$54,666/SDA~-$25,726/SDA) when compared to the traditional strategy. The optimal strategies within different willingness-to-pay (WTP) ranges were all precision screening strategies, and higher model sensitivities were adopted as WTP increased.

**Conclusions:**

Precision screening strategy for esophageal cancer based on risk stratification is more cost-effective than use of traditional screening strategy and has practical implications for esophageal cancer screening programs in China.

## Highlights

1. Well-performing risk prediction models for EC can achieve risk classification at both baseline screening and endoscopic surveillance in high-risk areas of China.
2. Precision screening strategies based on “two-step” individualized risk classification can largely avoid unnecessary screening for low-risk individuals, conserve health resources and increase the protection rate for cancer screening.

## Background

In China, EC(esophageal cancer) ranks as the sixth most frequent cancer and is the fourth most frequent cause of cancer death. Prognosis is often poor due to the fact a diagnosis is usually made at a late stage (1). No main cause of EC has been found which would allow primary prevention, and efforts are therefore focused on secondary prevention (2). Evidence from observational cohorts and nonrandomized controlled trials has shown that EC-specific mortality can be lowered by 66% (3, 4), and the 5-year survival rate can be increased from less than 30% to 80% through early screening (5, 6). Lugol’s iodine staining chromo-endoscopy is the gold standard technique for identifying high-grade squamous dysplasia and ESCC, and population-level endoscopic screening has been ongoing in high-risk areas of China since 2000 (7–9). By the end of 2018, over 2.16 million chromo-endoscopies had been performed, and over 34 thousand patients with malignant lesions had been diagnosed. More than 70% of the esophageal cancers which were detected were early stage (10).

For decades endoscopic screening for EC in China has adopted traditional “universal screening” for residents in high-risk communities (11–13). After initial screening, “pathology-based surveillance” recommended by expert consensus calls for endoscopic re-examination every 1 and 3 years for patients diagnosed with moderate and mild dysplasia respectively. No re-examination was offered for nondysplastic diagnoses (14). However, overscreening and insufficient diagnosis have become notable problems with this current screening modality due to the low absolute prevalence and high interpersonal heterogeneity of EC. According to recent studies, a significant degree of overscreening has occurred in universal endoscopic screening in China (15). Moreover, pathology-based surveillance has resulted in insufficient diagnosis or in missed SDA cases which had progressed (16). Precision screening strategies for ESCC based on efficient risk stratification are therefore needed to enable accurate identification of high-risk subgroups in target populations and to better facilitate the allocation of health resources (14, 17–19).

In this context, we propose a first “two-step” precision screening modality for ESCC, using two risk prediction models which performed well that were derived from the ESECC trial (16, 20, 21). First, before baseline screening, a model evaluating individual risk for “currently carrying malignant lesions in the esophagus” was adopted to identify high-risk subgroups, thus appreciably reducing the number of unnecessary endoscopies at baseline (21). After initial screening, a risk prediction model based on multifarious factors, including pathologic diagnosis and iodine staining status at baseline screening, was then applied to evaluate the progression risk of precursor lesions to malignant lesions. This notably increased the protection rate by surveillance for SDA cases that had been missed with traditional pathology-based surveillance which had progressed (16).

In this study, we aim to evaluate the cost-effectiveness of this “two-step” model-based precision screening modality as compared to the currently used universal screening modality based on a large population-level screening cohort. This study provides real-world evidence for the reform of esophageal cancer screening strategies in China.

## Methods

### Study platform

In January 2012, the ESECC randomized controlled trial (Clinical trial: NCT01688908) was initiated in Hua County, Henan Province, to evaluate the efficacy and cost-effectiveness of endoscopic screening for esophageal cancer in China (13). Residents from 668 target villages aged 45-69 years without a history of cancer or an endoscopic examination within 5 years were randomly selected and allocated to the screening arm or control arm of the study at a ratio of 1:1 (334 villages in each arm). Subjects in the screening arm were invited to undergo a standard endoscopic examination and biopsy with iodine staining, and then received both passive follow-up (linkage to health insurance reimbursement claims data to identify incident cancer cases) and active follow-up (door-to-door interviews) to collect vital events including the onset of cancer. The sensitivity of identifying incident cancer cases in the ESECC trial was estimated to be more than 95.6% (22). Subjects who were either biopsied due to visualization of unstained areas of mucosa, or diagnosed with mild or moderate dysplasia from the focal or standard sites at baseline examination in the screening arm, were invited to participate in follow-up surveillance, consisting of an individualized endoscopic re-examination carried out from May 2017 to November 2017 (16). In this study, a total of 15037 individuals from the screening arm of the ESECC trial who had received standard endoscopic examinations and provided adequate data on ESCC-related predictors (such as age, gender, Body Mass Index, ESCC family history, etc.) for risk evaluation were included in our modeling simulations to investigate the cost-effectiveness of precision screening strategies.

### Screening strategies and model building

**Figure 1:**
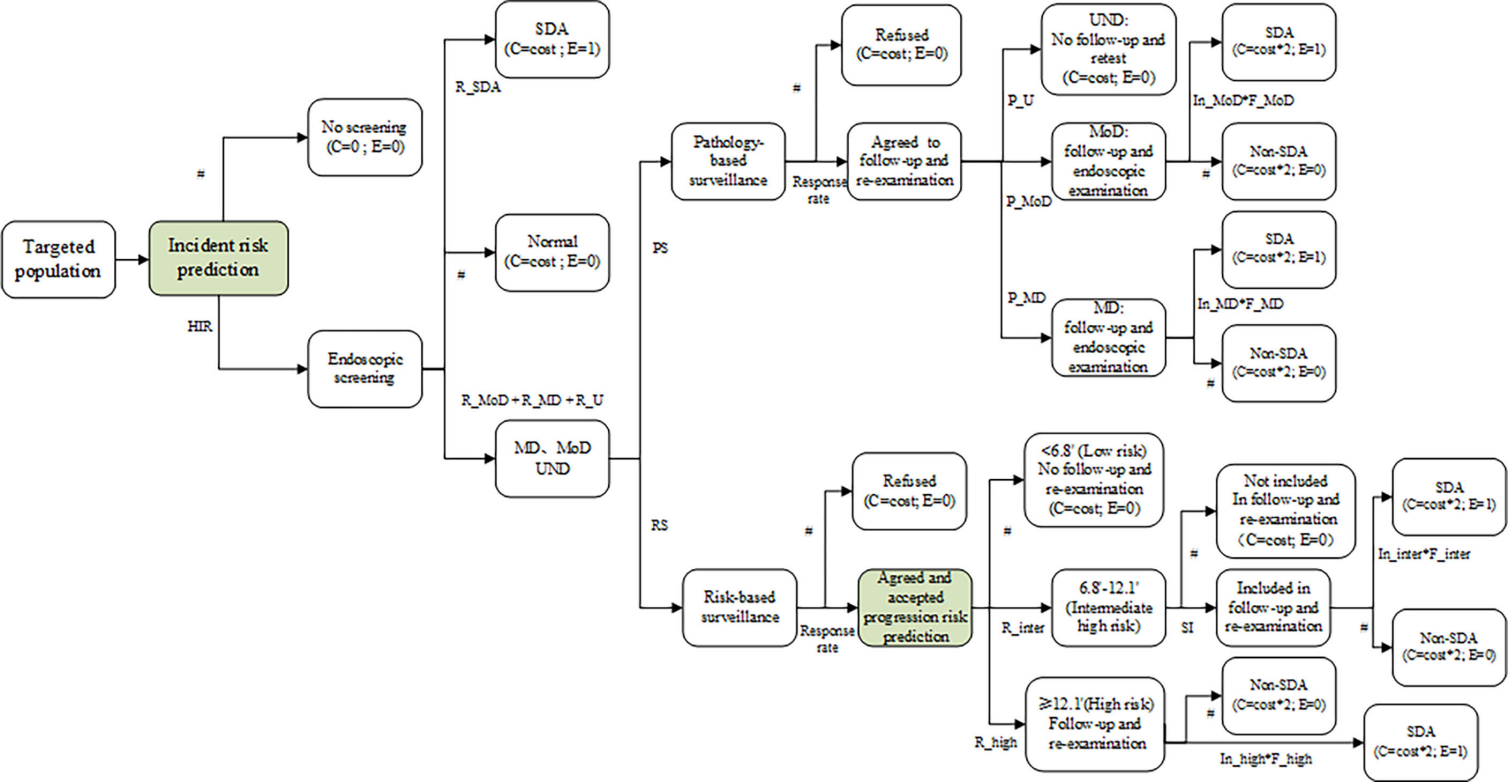
Structure of decision tree model for esophageal cancer screening strategies. HIR, High Incident-risk Rate in the prediction model at baseline according to adopted cutoff value and sensitivity (“1” refers to not using prediction model and screening for all enrolled participants at baseline). R_SDA, Detection rate of SDA cases for ESCC in the initial examination. R_MoD, Detection rate of Moderate dysplasia cases in the initial examination. R_MD, Detection rate of Mild dysplasia cases in the initial examination. R_U, Detection rate of subjects with visualization of unstained areas and non-dysplasia pathology diagnosis in the initial examination. PS, Adopting pathology-based surveillance. “0” refers to negative and “1” refers to positive. P_MD, Proportion of subjects with a diagnosis of Mild Dysplasia among all subjects with a diagnosis of MD, MoD or U. P_MoD, Proportion of subjects with a diagnosis of Moderate Dysplasia among all subjects with a diagnosis of MD, MoD or U. P_U, Proportion of subjects with a diagnosis of Unstaining and non-dysplasia among all subjects with a diagnosis of MD, MoD or U. In_MoD, Incidence rate (per person-year) of SDA cases in the surveillance among subjects with a diagnosis of Moderate Dysplasia. (“—” refers to no pathology-based surveillance adopted); F_MoD, Average follow-up interval (years) between baseline screening and re-examination for subjects with a diagnosis of Moderate Dysplasia. In_MD, Incidence rate (per person-year) of SDA cases in the surveillance among subjects with a diagnosis of Mild Dysplasia. (“—” refers to no pathology-based surveillance adopted); F_MD, Average follow-up interval (years) between baseline screening and re-examination for subjects with a diagnosis of Mild Dysplasia. RS, Adopting Risk-based Surveillance. “0” refers to negative and “1” refers to positive. SI, Surveillance for Intermediate high-risk subjects. “0” refers to negative and “1” refers to positive. (“—” refers to no risk-based surveillance adopted); R_high, Rate of high progression risk. (“—” refers to no risk-based surveillance adopted); R_inter, Rate of intermediate-high risk. (“—” refers to no risk-based surveillance adopted); In_high, Incidence rate (per person-year) of SDA cases in the surveillance among subjects with high progression risk. (“—” refers to no risk-based surveillance adopted); F_high, Average follow-up interval (years) between baseline screening and re-examination for subjects with high progression risk. In_inter, Incidence rate (per person-year) of SDA cases in the surveillance among subjects with intermediate high progression risk. (“—” refers to no risk-based surveillance adopted); F_inter, Average follow-up interval (years) between baseline screening and re-examination for subjects with intermediate high progression risk.

For brevity, the model building for all strategies were illustrated in **Figure 1**. The screening arm of the ESECC trial adopted the traditional strategy of implementing universal baseline screening and pathology-based surveillance in real-world intervention, for which related parameters were set (HIR=1; PS=1; RS=0) to open and close specific branches. As for the precision screening strategies (PS=0; RS=1), risk stratifications were designed to be realized with the application of a diagnostic model to identify prevalent malignant lesions in the esophagus, together with a prognostic model to predict the risk of progression of precancerous lesions (referring to the two green boxes in **Figure 1**). These two models had been developed previously based on the initial screening and follow-up of the participants in the screening arm of the ESECC trial (16, 21). First, before baseline screening, an evaluation of risk of onset for ESCC and its malignant lesions could be performed for each subject by calculating a specific risk score, which had been previously generated from the diagnostic model using individual information. We then assumed that only individuals with risk scores higher than the cutoff would be invited to undergo endoscopic examination. With a selected sensitivity of 100% (preset cutoff thresholds of 0.0007531 and 0.0049615 for ≤60-year and >60-year old age groups respectively) and 80% (preset cutoff thresholds of 0.0037069 and 0.0102338 for ≤60-year and >60-year old age groups respectively), this diagnostic model avoids 27% and 70% of endoscopies at the two sensitivities respectively in the ≤60-year age group, and avoids 9% and 40% of endoscopies respectively in the >60-year age group (21). After baseline endoscopic examination, a progression risk assessment was performed for patients in whom precancerous lesions were identified, including lesions pathologically diagnosed as mild or moderate dysplasia, and lesions with unstained areas. Similarly, progression risk scores for these patients were derived from the prognostic model that had been established based on age, pathologic diagnosis, BMI, and index of staining abnormalities (16). Using quartiles of risk scores as cutoff points, all subjects under evaluation were then divided into four subgroups with decreasing progression risk levels (high, intermediate-high, intermediate, and low; with corresponding risk score ranges of ≤4.0, 4.1~6.7, 6.8~12.0, and ≥12.1) (16). This model-based precision endoscopic surveillance had been proven to be able to increase the accuracy of predicting progression from 70% using the pathologic diagnosis alone to nearly 90%, and yielded protection for the missing 40% of SDA cases which had progressed by including them in surveillance (16).

For each strategy, cost and effectiveness were estimated in the <60-year group (9980 participants) and the ≥60-year group (5057 participants) respectively and then summed up as the total cost and effectiveness of the strategy. To evaluate precision screening strategies with varied combinations of risk-stratification capability, different risk thresholds were selected for both models. Two model sensitivities (100% or 80%) were first utilized for risk stratification at baseline screening. For subsequent surveillance (endoscopic re-examination). Two alternatives were designed including enrolling subjects with high progression risk or high and intermediate-high progression risk for endoscopic re-examination. The risk scores for prevalent cancer and progression of precancerous lesions for all 15073 participants included in this modeling study had already been calculated using the two previously derived models. In our simulation, each subject could be assigned to different screening interventions according to the risk thresholds which we adopted. The final status of each participant was therefore determined once the two risk thresholds were decided, and transmission rates of the target population could be obtained. Eventually, a total of 4 (2*2) precision screening strategies were constructed through permutation and combination of risk thresholds at each of the two steps (baseline screening and endoscopic surveillance) as follows:

1) **precision strategy_1:** model sensitivity of 100% at baseline screening, and surveillance for subgroups with high and intermediate high risk of progression
2) **precision strategy_2:** model sensitivity of 100% at baseline screening, and surveillance for subgroups with high risk of progression
3) **precision strategy_3:** model sensitivity of 80% at baseline screening and surveillance for subgroups with high and intermediate high risk of progression
4) **precision strategy_4:** model sensitivity of 80% at baseline screening and surveillance for subgroups with high risk of progression

### Cost and effectiveness

This study focuses on the effectiveness of risk stratification for improving the detection rate of a screening program from the perspective of the public payer. All related costs generated during the screening intervention (referred to as screening costs below) were considered, such as costs for drugs, equipment, and administrative costs for the screening program. The average cost of $134 (conversed to the year 2018) per person-time for endoscopic examination had been reported in our previous research and directly used in this modeling study (baseline or re-examination) (23). A discount rate of 3% had been adopted in the initial estimation of the average screening cost, and no discounting was performed for costs and outcomes in this decision tree analysis. A response rate of 0.661 from real-world evidence was adopted for re-examination acceptance (16). The response rate was assumed to be the same in pathology-based and risk-based surveillance since the standards for participant selection were unlikely to influence the willingness for acceptance. Risk assessment could be implemented using information available from existing questionnaires and baseline screening diagnosis, the cost of which had been mostly covered by the screening cost. Costs for risk assessment were therefore assumed to be zero in our precision screening strategies. The effectiveness evaluated in this study was defined as the total number of SDA cases detected in baseline screening and endoscopic surveillance in the screening cohort of the ESECC trial under varied precision screening strategies. The control arm was not considered in this study, and cost-effectiveness was compared for different screening strategies designed for the screening arm. In real-world practice, a total of 163 SDA cases were identified in the screening arm, including those detected in the universal baseline screening and in the one-time re-examination, as well as those diagnosed clinically and reported during follow-up. Given this fact, we took 163 as the maximum number of SDA cases that were supposed to be detected during the study period, and a protection rate was calculated for each strategy as the ratio of effectiveness to the 163 SDA cases.

### Cost-effectiveness evaluation

A decision tree model was developed (using the “heemod” package in R3.6.2) to project the costs and effectiveness of four precision strategies and the traditional strategy, and the analysis was reported according to the Consolidated Health Economic Evaluation Reporting Standards (CHEERS) statement (24). For each strategy, a cohort of 15037 residents from the screening arm of the ESECC trial were entered into the model, and some were invited to baseline screening and endoscopic surveillance decided by preset risk thresholds, resulting in differing final status as illustrated in **Figure 1**.

The base-case analysis a comparison of the traditional screening strategy and other precision screening strategies. All of these strategies have the same structure (**Figure 1**), but have different transition probabilities obtained from our previous studies where the two prediction models were established. (**Table 1**). The incremental cost for detecting one additional SDA case was estimated in two ways, using the previous less costly strategy and the traditional strategy as reference respectively.

**Table 1:** Age-stratified parameters and reference sources of two-step precision screening strategies in the decision tree model for esophageal cancer screening in high-risk areas of China.

Age	Strategy	HIR^a^	R_SDA^b^	R_MoD^c^	R_MD^d^	R_U^e^	PS^f^	P_MD^g^	P_MoD^h^	P_U^i^	In_MoD^j^	F_MoD^k^	In_MD^l^	F_MD^m^	RS^n^	SI^°^	R_high^p^	R_inter^q^	In_high^r^	F_high^s^	In_inter^t^	F_inter^u^
<60	Traditional	1.0000	0.0038	0.0008	0.0064	0.0416	1	0.1311	0.0164	0.8525	0.0665	4.358	0.0286	4.358	0	0	——	——	——	——	——	——
<60	Precision 1	0.7440	0.0051	0.0011	0.0081	0.0474	0	0.1431	0.0194	0.8375	——	——	——	——	1	1	0.1619	0.2571	0.0377	4.253	0.0093	4.253
<60	Precision 2	0.7440	0.0051	0.0011	0.0081	0.0474	0	0.1431	0.0194	0.8375	——	——	——	——	1	0	0.1619	0.2571	0.0377	4.253	——	——
<60	Precision 3	0.3067	0.0098	0.0023	0.0108	0.0627	0	0.1425	0.0303	0.8272	——	——	——	——	1	1	0.2112	0.2802	0.0377	4.253	0.0093	4.253
<60	Precision4	0.3067	0.0098	0.0023	0.0108	0.0627	0	0.1425	0.0303	0.8272	——	——	——	——	1	0	0.2112	0.2802	0.0377	4.253	——	——
≥60	Traditional	1.0000	0.0154	0.0053	0.0194	0.0874	1	0.1731	0.0473	0.7797	0.0665	4.358	0.0286	4.358	0	0	——	——	——	——	——	——
≥60	Precision 1	0.9324	0.0165	0.0055	0.0197	0.0884	0	0.1734	0.0484	0.7782	——	——	——	——	1	1	0.3563	0.2724	0.0377	4.253	0.0093	4.253
≥60	Precision 2	0.9324	0.0165	0.0055	0.0197	0.0884	0	0.1734	0.0484	0.7782	——	——	——	——	1	0	0.3563	0.2724	0.0377	4.253	——	——
≥60	Precision 3	0.5642	0.0217	0.0070	0.0210	0.0922	0	0.1747	0.0582	0.7671	——	——	——	——	1	1	0.3819	0.2595	0.0377	4.253	0.0093	4.253
≥60	Precision 4	0.5642	0.0217	0.0070	0.0210	0.0922	0	0.1747	0.0582	0.7671	——	——	——	——	1	0	0.3819	0.2595	0.0377	4.253	——	——
**Reference sources**		(21)	(21)	(21)	(21)	(21)	**—**	(16)	(16)	(16)	(16)	(16)	(16)	(16)	**—**	**—**	(16)	(16)	(16)	(16)	(16)	(16)

**^a^** HIR, High Incident-risk Rate in the prediction model at baseline according to adopted cutoff value and sensitivity (“1” refers to not using prediction model and screening for all enrolled participants at baseline).

**^b^** R_SDA, Detection rate of SDA cases for ESCC in the initial examination.

**^c^** R_MoD: Detection rate of Moderate dysplasia cases in the initial examination.

**^d^** R_MD: Detection rate of Mild dysplasia cases in the initial examination.

**^e^** R_U, Detection rate of subjects with visualization of unstained areas and non-dysplasia pathology diagnosis in the initial examination.

**^f^** PS, Adopting pathology-based surveillance. “0” refers to negative and “1” refers to positive.

**^g^** P_MD, Proportion of subjects with a diagnosis of Mild Dysplasia among all subjects with a diagnosis of MD, MoD or U.

**^h^** P_MoD, Proportion of subjects with a diagnosis of Moderate Dysplasia among all subjects with a diagnosis of MD, MoD or U.

**^i^** P_U, Proportion of subjects with a diagnosis of Unstaining and non-dysplasia among all subjects with a diagnosis of MD, MoD or U.

**^j^** In_MoD, Incidence rate (per person year) of SDA cases in the surveillance among subjects with a diagnosis of Moderate Dysplasia. (“—” refers to no pathology-based surveillance adopted).

**^k^** F_MoD, Average follow-up interval (years) between baseline screening and re-examination for subjects with a diagnosis of Moderate Dysplasia.

**^l^** In_MD: Incidence rate (per person year) of SDA cases in the surveillance among subjects with a diagnosis of Mild Dysplasia. (“—” refers to no pathology-based surveillance adopted).

**^m^** F_MD, Average follow-up interval (years) between baseline screening and re-examination for subjects with a diagnosis of Mild Dysplasia.

**^n^** RS, Adopting Risk-based Surveillance. “0” refers to negative and “1” refers to positive.

**^°^** SI: Surveillance for Intermediate high-risk subjects. “0” refers to negative and “1” refers to positive. (“—” refers to no risk-based surveillance adopted).

**^p^** R_high, Rate of high progression risk. (“—” refers to no risk-based surveillance adopted).

**^q^** R_inter, Rate of intermediate-high risk. (“—” refers to no risk-based surveillance adopted).

**^r^** In_high, Incidence rate (per person year) of SDA cases in the surveillance among subjects with high progression risk. (“—” refers to no risk-based surveillance adopted).

**^s^** F_high, Average follow-up interval (years) between baseline screening and re-examination for subjects with high progression risk.

**^t^** In_inter, Incidence rate (per person year) of SDA cases in the surveillance among subjects with intermediate high progression risk. (“—” refers to no risk-based surveillance adopted)

**^u^** F_inter, Average follow-up interval (years) between baseline screening and re-examination for subjects with intermediate high progression risk.

### Sensitivity analysis

Probabilistic sensitivity analysis was conducted using Monte Carlo simulations involving random sampling from distributions assigned to parameters, including the average screening cost of endoscopic examination, response rate of subjects in the re-examination (proportion of participants who accepted endoscopic re-examination upon invitation), follow-up interval for individuals at high-risk and intermediate-high-risk after baseline screening, and the follow-up interval for mild dysplasia and moderate dysplasia individuals after baseline screening, to investigate simultaneous effects of parameter uncertainty of the base-case results. Compared with the same follow-up interval (4.358 years) set for individuals with mild dysplasia and moderate dysplasia in base case analysis, gamma distributions with different mean values (1 year for mild dysplasia or high progression risk individuals; 3 years for moderate dysplasia or intermediate-high progression risk individuals) were assigned to individuals with varied progression risks in probabilistic sensitivity analysis in consideration of recommendations by expert consensus. (**Supplementary Table 1** in Online Resource 1) One-way sensitivity analysis for the same set of parameters above were also performed for cost-effective strategies. Based on simulations of 1000 iterations, cost-effectiveness acceptability curves (CEACs) and cost-effectiveness acceptability frontiers (CEAFs) were drawn to present the optimal decisions under different WTP ranges, together with the uncertainty level associated with each optimal decision. Unlike the “two-step” precision strategy_1~4 where risk-stratification was conducted at both baseline screening and at endoscopic surveillance, strategies adopting “one-step” risk-stratification at either baseline screening or subsequent endoscopic surveillance (precision strategy_5~8) were also designed and evaluated in the supplementary analysis. Precision strategy_5/6 adopted only model-based baseline screening with sensitivities of 100%/80% respectively. Precision strategies_7/8 adopted only model-based endoscopic surveillance for participants with high and intermediate high progression risk/high progression risk. Parameters for precision strategies_5~8 are presented in **Supplementary Table 2** (Online Resource 1).

## Results

### Cost-effectiveness of screening strategies

In the base case analysis, all precision strategies showed better performance with considerably lower average costs for detecting one SDA case as compared to the traditional strategy, ($7,148~$11,537 *vs*. $14,944) (**Table 2**). When the model sensitivity was set as 100% at baseline, precision strategy_1 and precision strategy_2 achieved higher effectiveness (143~150 *vs.* 136) and correspondingly higher protection rates (87·7%~92·0% *vs*. 83·4%) at much lower costs ($1,649,727~$1,672,221 *vs.* $2,032,386) than the traditional strategy. When a lower model sensitivity of 80% was selected for precision strategy_3 and precision strategy_4 at baseline, total costs decreased sharply to only 40% ($808,420~$822,061 *vs*. $2,032,386), while effectiveness remained at approximately 85% (111~115 *vs*. 136) compared to the traditional strategy, resulting in the lowest average screening costs of nearly half ($7,148~$7,283 *vs*. $14,944).

**Table 2:** Cost-effectiveness of esophageal cancer screening strategies from the ESECC trial in high-risk areas of China.

Strategy	Baseline enrollment	Surveillance enrollment	Cost(USD)	Effectiveness(SDA)	Cost/Effectiveness	Protection rate^a^	ICER (per SDA detected)	
							*vs.* traditional screening	*vs.* previous strategy^b^
Precision screening_4	Model-based (Sensitivity of 80%)	Model-based (High progression risk)	808,420	111	7,283	68.1%	48,959	N/A
Precision screening_3	Model-based (Sensitivity of 80%)	Model-based (High and intermediate high progression risk)	822,061	115	7,148	70.6%	57,635	$ 3,410
Precision screening_2	Model-based (Sensitivity of 100%)	Model-based (High progression risk)	1,649,727	143	11,537	87.7%	-54,666	**Extended Dominated**
Precision screening_1	Model-based (Sensitivity of 100%)	Model-based (High and intermediate high progression risk)	1,672,221	150	11,148	92.0%	-25,726	$ 24,290
Traditional screening	Universal screening	Pathology-based	2,032,386	136	14,944	83.4%	**N/A**	**Dominated**

ESECC, the Endoscopic Screening for Esophageal Cancer in China (ESECC) randomized controlled trial (Clinical trial: NCT01688908); USD, US Dollars; ICER, Incremental Cost Effectiveness Ratio; SDA, Severe Dysplasia and Above lesions for esophageal squamous cell carcinoma.

**^a^** Protection rate was calculated for each strategy as the ratio of effectiveness to the 163 SDA cases detected in the universal baseline screening and in a large-scale re-examination as well as cases identified through follow-up from the screening cohort of the ESECC trial in the real world.

^b^Previous strategy refers to previous less costly strategy that is not dominated or extended dominated.

ICERs were calculated across all strategies and the cost-effectiveness plane is presented in **Supplementary Figure 1** (Online Resource 1). The cost-effectiveness frontier consisted of 3 strategies: precision screening_4, precision screening_3, and precision screening_1, which generated increasing ICERs of $3,410 and $24,290 (**Table 2**). When compared with the traditional strategy, strategy precision screening_2 and strategy precision screening_1 had negative ICERs (-$54,666/SDA~-$25,726/SDA), indicating increased effectiveness at lower cost.

Comparing precision strategies with the same model sensitivity set at baseline, precision strategy_3 had a relatively lower average cost than precision strategy_4 ($7,148 *vs*. $7,283) due to the higher protection rate (70·6% *vs*. 68·1%) from a broader range of surveillance (high and intermediate high progression risk) and slightly increased total cost, which is the same for comparison of precision strategy_1 and precision strategy_2.

### Sensitivity analysis

**Figure 2:**
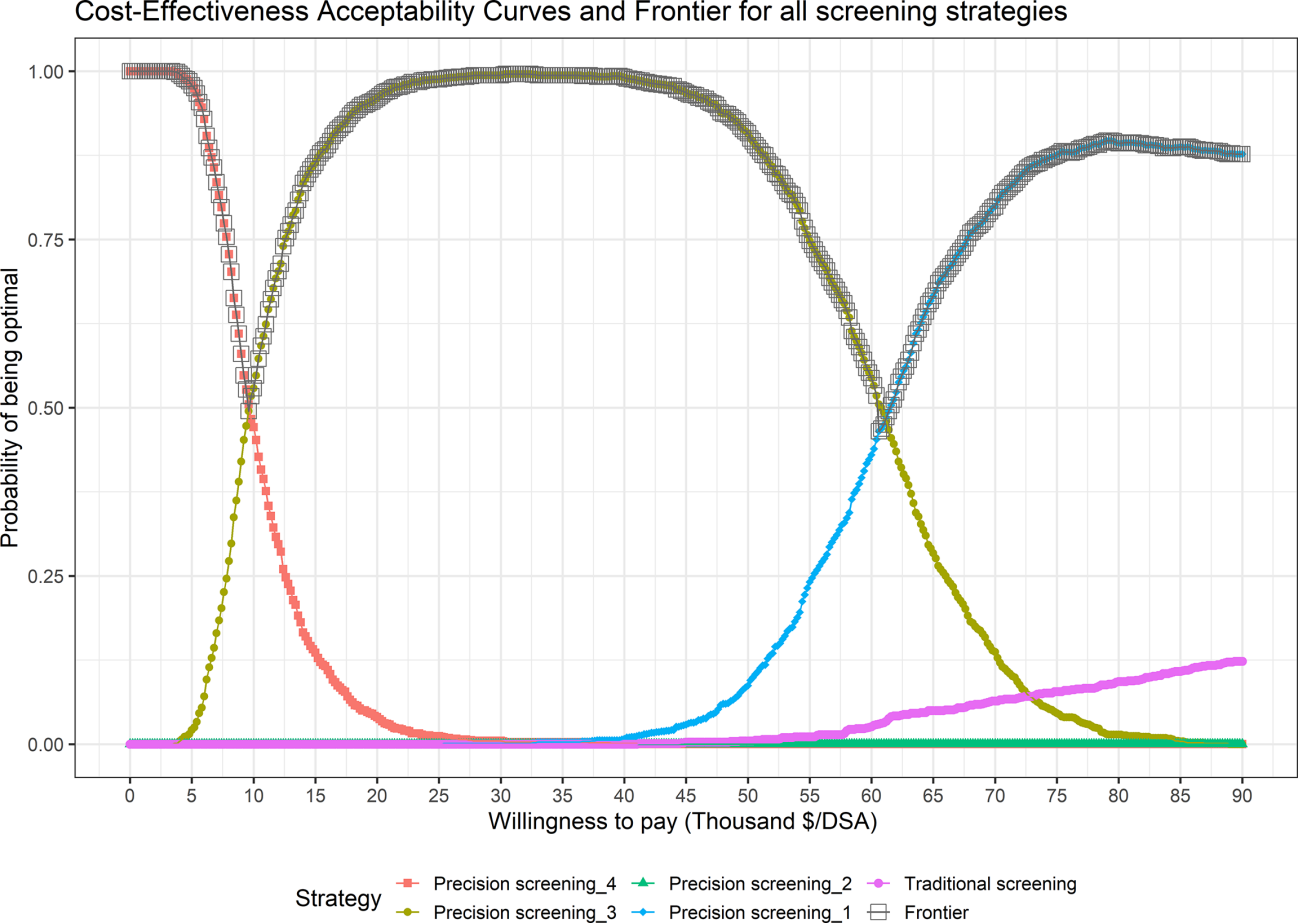
Cost-Effectiveness Acceptability Curves (CEACs) and Frontier (CEAF) in the Probability Sensitivity Analysis (PSA) of different screening strategies.

**Figure 2** shows cost-effectiveness acceptability curves (CEACs) and a cost-effectiveness acceptability frontier (CEAF) presenting optimal strategies with probabilities of being cost-effective under different WTPs. For resource-limited areas with a WTP threshold of less than $9,465/SDA, precision strategy_4 (80%; surveillance for high progression risk) which incurred the lowest total screening cost was the optimal choice. When WTP varied within a range of $9,465~$60,478/SDA, precision strategy_3 (80%; surveillance for high and intermediate high progression risk) became the preferred screening strategy. When WTP became greater than $60,478/SDA, precision strategy_1 (100%; surveillance for high and intermediate high progression risk) with the lowest average cost and the highest protection rate remained the dominant strategy with a probability of 100% of being optimal.

In one-way sensitivity analysis for cost-effective strategies (**Supplementary Figure 2** in Online Resource 1), the ICER of precision screening_3 to precision screening_4 was sensitive to the surveillance interval for intermediate-high progression risk individuals. The ICER of precision screening_1 to precision screening_3 was sensitive to average screening cost, the response rate for re-examination, and surveillance intervals for high and intermediate-high progression risk individuals.

When comparing the traditional strategy with “one-step” precision screening strategies (precision strategy_5~8), all three cost-effective strategies were precision screening strategies, including precision strategies_6 & 5 applying model-based baseline screening at a sensitivity of 80% and 100%, and precision strategy_7 applying model-based surveillance for individuals with high and intermediate high progression risk (**Table 3**).

**Table 3:** Cost-effectiveness of one-step precision strategies for esophageal cancer screening in high-risk area of China.

Strategy	Baseline enrollment	Surveillance enrollment	Cost(USD)	Effectiveness(SDA)	Cost/Effectiveness	Protection rate^a^	ICER (per SDA detected)	
							*vs.* traditional screening	*vs.* previous strategy^b^
Precision screening_6	Model-based (Sensitivity of 80%)	Pathology-based	803,107	105	7,649	64.4%	39,654	**N/A**
Precision screening_5	Model-based (Sensitivity of 100%)	Pathology-based	1,643,371	135	12,173	82.8%	389,015	$ 28,009
Traditional screening	Universal screening	Pathology-based	2,032,386	136	14,944	83.4%	**N/A**	**Dominated**
Precision screening_8	Universal screening	Model-based (High progression risk)	2,038,867	144	14,159	88.3%	810	**Extended Dominated**
Precision screening_7	Universal screening	Model-based (High and intermediate high progression risk)	2,062,778	152	13,571	93.3%	1,900	$ 24,671

ESECC, the Endoscopic Screening for Esophageal Cancer in China (ESECC) randomized controlled trial (Clinical trial: NCT01688908); USD, US Dollars; ICER, Incremental Cost Effectiveness Ratio; SDA, Severe Dysplasia and Above lesions for esophageal squamous cell carcinoma.

**^a^** Protection rate was calculated for each strategy as the ratio of effectiveness to the 163 SDA cases detected in the universal baseline screening and a large-scale re-examination as well as cases identified through follow-up from the screening cohort of the ESECC trial in the real world.

^b^Previous strategy refers to previous less costly strategy that is not dominated or extended dominated.

## Discussion

Secondary prevention is currently an important means of control for ESCC worldwide. However, conventional universal endoscopic screening at baseline together with pathology-based surveillance would qualify a large number of low-risk individuals for screening and re-examination, exposing them to an unnecessary invasive examination which might result in increased budgetary pressure. More importantly, insufficient diagnosis and inadequate re-examination resulting from conventional pathology-based surveillance may impair the protection ability of the screening by mistakenly excluding individuals at high risk of progression from surveillance. Several population-level endoscopic screening programs for ESCC have been carried out in high-risk areas of China over the past several decades, and the screening strategy currently employed urgently calls for refinement to achieve better allocation of health resources. Concepts for precision screening modalities based on two risk prediction models for ESCC generated from a real-world screening trial have been proposed (20). In this study, health economic evaluations were conducted to compare the cost-effectiveness of risk-stratification-based precision screening modalities with traditional screening practices. All three cost-effective strategies were precision screening strategies with much lower average costs for detecting one patient with a malignant lesion in the esophagus. When using the traditional strategy as the reference strategy, precision strategy_1 (100%; surveillance for high and intermediate high progression risk) and precision strategy_2 (100%; surveillance for high progression risk) obtained negative ICERs with higher effectiveness and lower total costs as compared to traditional strategy. To the best of our knowledge, this is the first study to evaluate the cost-effectiveness of “two-step” model-based precision endoscopic screening modalities for ESCC in China.

Risk stratification tools with good performance are a crucial precondition for excellence in personalized cancer screening programs in view of the challenges confronted in the use of conventional screening strategy. It has been reported that up to 73·7% of all participants in a screening program accepted pathologic biopsy at baseline because of abnormal iodine staining. However, less than 3% of those who accepted pathologic biopsy were diagnosed with SDA (15). With the use of a model which predicts the risk of “currently carrying malignant lesions of ESCC” at baseline screening, 21% of endoscopic examinations could be avoided at a model sensitivity of 100%, and a higher proportion of 60% could be avoided at a sensitivity of 80% (21). This is of significant value when resources for endoscopic screening are extremely limited and identifying greater numbers of cancer patients at a lower average cost is the highest priority. Since examinations at baseline account for a large proportion of the total workload, risk classification at this step can play a key role in total cost saving. Second, for conventional pathology-based endoscopic surveillance, results from over 8·5 years of follow-up showed that only 1·4% of mild dysplasia and 4·5% of moderate dysplasia progressed to ESCC (15). Moreover, in another study, after a median follow-up interval of 4·2 years, over 40% of SDA cases which had progressed were assigned a nondysplasia pathologic diagnosis. These cases would have been excluded from pathology-based surveillance (16). Through risk stratification based on a model which considers comprehensive risk factors to a greater extent than baseline pathologic diagnosis, the protection rate could be improved with just a slight increase in the endoscopic surveillance workload. Moreover, the protection rate might be further increased if a greater effort was invested in achieving a higher response rate during invitation and mobilization for re-examination, as this model also performs well among those who did not attend endoscopic re-examination (16). It is noteworthy that in comparison to other risk prediction models which merely focus on esophageal cancer cases from observational cohorts (25), our precision screening modalities which are based on real-world screening trials also gave attention to outcomes for malignant precancerous ESCC lesions such as severe dysplasia. This was of greater practical value in early detection and early treatment for cancer prevention. As far as we are concerned, the “two-step” precision screening modalities and risk prediction models are the most complete and precise risk classification tools for ESCC screening in China to date and are easy to implement in practice.

Precision screening strategies with different combinations of risk thresholds at baseline and under surveillance showed varied cost and effectiveness, demonstrating the balance of cost savings and improvement of effectiveness under the joint control of risk stratification at baseline enrollment and endoscopic surveillance. Compared to traditional strategy, precision strategy_1 and precision strategy_2 with a model sensitivity of 100% at baseline could not only achieve an approximately 20% total cost savings (1·6 *vs.* 2 million dollars) but also yield higher protection rates (87·7%~92·0% *vs.* 83·4%) from model-based surveillance, contributing to outstanding resource utilization with negative ICERs. When a lower sensitivity of 80% was selected at baseline for precision strategy_3 and precision strategy_4, the effect on cost savings (0·8 *vs.* 2 million) far exceeded the loss of total effectiveness (111~115 *vs.* 136) due to the enrollment of fewer participants. The lowest average cost for detecting one SDA case was therefore achieved with precision strategy_3 ($7,148). Supplementary analysis of precision strategy_5~8 with an application of precision screening at either step provided further information, demonstrating that risk stratification at baseline screening and endoscopic surveillance may contribute to cost savings and improved effectiveness respectively.

According to the results of sensitivity analysis, precision strategy_1 detected the most SDA cases of all strategies, maintained a probability of around 100%, and was the optimal strategy when WTP was greater than 24.29 thousand dollars, displaying absolute dominance over other precision strategies. In underdeveloped areas with WTP ranges of 0~3.41 and 3.41~24.29 thousand dollars, the recommended choice of strategy is precision strategy_3 and precision strategy_4 respectively, with a lower sensitivity of 80% at baseline enrollment and corresponding total screening costs which are much lower. Although a specific WTP threshold was not adopted in this study, the optimal strategy and its uncertainty under different WTP ranges presented by the CEAF curve could still provide implications for precision screening strategy formulation in areas of China with differing capacities to pay. However, we must be careful about the theoretical recommendation of a lower model sensitivity in real-world community-based screening practice. On one hand, a lower sensitivity may result in a higher risk threshold, and fewer residents would enroll in the screening program compared with a sensitivity of 100% or traditional universal screening, which might cause social problems related to satisfaction with the program, or with ethics and health equities. This in particular might occur in less developed communities where cancer screening provided by the government is more likely to be considered as public welfare. On the other hand, we should keep in mind that risk assessment scores lower than the preset cutoff do not guarantee an absolute low risk of ESCC from an individualized point of view. It is therefore of great importance to provide clear informed consent and health education during the implementation of risk-stratified precision cancer screening.

In previous studies of precision screening for ESCC, a modeling study by Xia et al. reported that risk-stratified endoscopic screening for esophageal cancer is more cost-effective than universal screening or absence of screening based on simulated populations and parameters compiled from several sources (26). However, this study modeled a screening modality which was carried out only once in a lifetime, and this is not in keeping with current screening practice. Moreover, this approach had been repeatedly proven to be less cost-effective than screening involving follow-up and re-examination in other modeling studies (27–29). High-quality comparative evaluations based on real-world screening and data for comprehensive precision screening modalities regarding baseline screening as well as following endoscopic surveillance are urgently needed.

Compared to other modeling studies, the study subjects, screening cost, health outcomes, construction of two risk prediction models, and the epidemiologic parameters of this research were all high-quality real-world data obtained from the ESECC screening cohort, in which local populations were well represented. In addition, with the application of two risk-stratification models which perform well, the precision screening strategies constructed in this study showed steady dominance over the traditional strategy under varied WTPs, providing practical guidance for policy-making under varying social development statuses.

This study has limitations. It was a single-center study, and the performance of a given precision screening strategy may not fit well in other populations with different characteristics. In addition, the short-term outcome of SDA cases in this study, which is also the primary appraisal indicator for screening programs by government departments, was only able to investigate cost-effectiveness with regard to the detection rate. The long-term value of risk-stratified screening intervention still requires systematic evaluation based on population-level randomized controlled trials, using life years or QALYs as effectiveness measurement. Such evidence from the ESECC trial will be reported in the near future.

## Conclusions

In comparison with traditional universal screening, precision screening strategies taking advantage of well-performing prediction models to achieve risk classification at baseline screening and endoscopic surveillance can largely avoid unnecessary screening for low-risk individuals, conserve health resources and increase the protection rate for cancer screening. This all shows great potential for improvement of the cost-effectiveness of ESCC cancer screening programs in undeveloped high-risk areas of China.

## Data availability statement

The datasets presented in this article are not readily available because the datasets used and/or analyzed during the current study are available from the corresponding author on reasonable request. Requests to access the datasets should be directed to zhonghuhe@foxmail.com.

## Ethics statement

The studies involving human participants were reviewed and approved by the Institutional Review Board of the Peking University School of Oncology, Beijing, China. The patients/participants provided their written informed consent to participate in this study.

## Author contributions

ZH and FXL contributed to the conception and design of the study. ML, CG, RX, FLL, ZL, YP, FFL, YL, HC, and ZH, contributed to the acquisition of data. FXL and ZH contributed to data analysis. YK, ZH, and FXL contributed to interpretation of data and checking results. YK, ZH, and FXL contributed to drafting the manuscript, which was reviewed and approved by all coauthors. All authors contributed to the article and approved the submitted version.

## Funding

This work was supported by the National Science & Technology Fundamental Resources Investigation Program of China (No. 2019FY101102), the National Key R&D Program of China (No. 2021YFC2500405), the National Natural Science Foundation of China (No. 82073626, 81773501), the BeijingTianjin-Hebei Basic Research Cooperation Project (No. J200016), the Beijing Nova Program (No. Z201100006820093) and the China Postdoctoral Science Foundation (Grant No. 2022M723289). Those involved in the funding of this study had no role in the study design, data collection, data analysis, data interpretation, or writing of the report. The corresponding authors had full access to all data in the study and had final responsibility for the decision to submit for publication.

## Acknowledgments

The authors thank all the following team members and collaborators for their contributions to the field work done for this study, including endoscopic examinations and pathologic diagnosis (in alphabetical order by last name and first name): Changqi Cao, Qiuju Deng, Dong Hang, Jingjing li, Shijie li, Xiang Li, Yan li, Zhihao Lu, Lin Shen, Na Shen, Haixing Wang, Hui Wang, Jing Wang, Xicheng Wang, Qi Wu, Yan Yan, Wenqing Yuan, Chanyuan Zhang, Chaoting Zhang, Xiaotian Zhang, and Jun Zhou from Peking University Cancer Hospital & Institute; Liping Duan, Kun Wang, Ye Wang, and Li Zhang from Peking University Third Hospital; Wanju Gao, Mei Guo, Anxiang Liu, Qianqian Meng, Haijun Yang, Jun Yang, Liheng Zhang, Lixin Zhang, and Sanshen Zhang from Anyang cancer Hospital, Henan Province; Yujie He, Shaojiang Lv, and Xiangqin Song from the People’s Hospital of Hua county, Henan Province; Xin Yang and Weiguo Xu from the North China University of Science and Technology Affiliated Hospital, Hebei Province; Zengchao Chen from Shandong Qianfoshan Hospital, Shandong Province. The authors would also like to thank the government of Anyang City and Hua County, the Health Commission of Anyang City and Hua County, Henan Province, and all the participants in the ESECC trial.

## Conflict of interest

The authors declare that the research was conducted in the absence of any commercial or financial relationships that could be construed as a potential conflict of interest.

## Publisher’s note

All claims expressed in this article are solely those of the authors and do not necessarily represent those of their affiliated organizations, or those of the publisher, the editors and the reviewers. Any product that may be evaluated in this article, or claim that may be made by its manufacturer, is not guaranteed or endorsed by the publisher.

## Abbreviations

EC, esophageal cancer; ESCC, esophageal squamous cell carcinoma; ESECC, Endoscopic Screening for Esophageal Cancer in China; SDA, severe dysplasia and above lesions in the esophagus; ICER, incremental cost-effectiveness ratio; CEAC, cost-effectiveness acceptability curve; CEAF, cost-effectiveness acceptability frontier; WTP, willingness to pay; PSA ,probability sensitivity analysis.
