# Supplementary material for: Cost-effectiveness of precision screening for esophageal cancer based on individualized risk stratification in China: Real-world evidence from the ESECC trial
Source: Front Oncol. 2022 Nov 30;12:1002693. doi: 10.3389/fonc.2022.1002693 (PMC9748682; doi:10.3389/fonc.2022.1002693)
Supplement: Supplementary file 1 [file DataSheet_1.docx]

**Supplementary Figure 1. Cost-effectiveness plane and cost-effectiveness frontier for all endoscopic screening strategies in the base case analysis**

**Supplementary Figure 2. Tornado diagrams for cost-effective strategies in one-way sensitivity analysis**

| **Supplementary Table 1. Parameter settings in** **sensitivity analysis.** | | |  |
| --- | --- | --- | --- |
| **Variable** | **Probabilistic sensitivity analysis** | | **One-way sensitivity analysis** |
|  | **Distribution type** | **Distribution parameters** |  |
| Unit cost for endoscopic screening | Gamma | Mean=134; SD=30 | (104, 168) |
| Response rate for endoscopic surveillance | Uniform | Min=0.50; max=1.00 | (0.5, 1.0) |
| Surveillance interval for high progression risk individuals | Gamma | Mean=1; SD=1 | (3.253, 5.253) |
| Surveillance interval for intermediate-high progression risk individuals | Gamma | Mean=3; SD=1 | (3.253, 5.253) |
| Surveillance interval for individuals with mild dysplasia | Gamma | Mean=3; SD=1 | (3.358, 5.358) |
| Surveillance interval for individuals with moderate dysplasia | Gamma | Mean=1; SD=1 | (3.358, 5.358) |

| **Supplementary Table 2. Age-stratified parameters and reference sources of two-step precision screening strategies in the decision tree model for esophageal cancer screening in high-risk area of China.** | | | | | | | | | | | | | | | | | | | | | | |
| --- | --- | --- | --- | --- | --- | --- | --- | --- | --- | --- | --- | --- | --- | --- | --- | --- | --- | --- | --- | --- | --- | --- |
| Age | Strategy | HIR ^a^ | R_SDA ^b^ | R_MoD ^c^ | R_MD ^d^ | R_U ^e^ | PS ^f^ | P_MD ^g^ | P_MoD ^h^ | P_U ^i^ | In_MoD ^j^ | F_MoD ^k^ | In_MD ^l^ | F_MD ^m^ | RS ^n^ | SI ^o^ | R_high ^p^ | R_inter ^q^ | In_high ^r^ | F_high ^s^ | In_inter  ^t^ | F_inter ^u^ |
| <60 | Precision 5 | 0.7440 | 0.0051 | 0.0011 | 0.0081 | 0.0474 | 1 | 0.1431 | 0.0194 | 0.8375 | 0.0665 | 4.358 | 0.0286 | 4.358 | 0 | 0 | —— | —— | —— | —— | —— | —— |
| <60 | Precision 6 | 0.3067 | 0.0098 | 0.0023 | 0.0108 | 0.0627 | 1 | 0.1425 | 0.0303 | 0.8272 | 0.0665 | 4.358 | 0.0286 | 4.358 | 0 | 0 | —— | —— | —— | —— | —— | —— |
| <60 | Precision 7 | 1.0000 | 0.0038 | 0.0008 | 0.0064 | 0.0416 | 0 | 0.1311 | 0.0164 | 0.8525 | —— | —— | —— | —— | 1 | 1 | 0.1437 | 0.2341 | 0.0377 | 4.253 | 0.0093 | 4.253 |
| <60 | Precision 8 | 1.0000 | 0.0038 | 0.0008 | 0.0064 | 0.0416 | 0 | 0.1311 | 0.0164 | 0.8525 | —— | —— | —— | —— | 1 | 0 | 0.1437 | 0.2341 | 0.0377 | 4.253 | —— | —— |
| ≥60 | Precision 5 | 0.9324 | 0.0165 | 0.0055 | 0.0197 | 0.0884 | 1 | 0.1734 | 0.0484 | 0.7782 | 0.0665 | 4.358 | 0.0286 | 4.358 | 0 | 0 | —— | —— | —— | —— | —— | —— |
| ≥60 | Precision 6 | 0.5642 | 0.0217 | 0.0070 | 0.0210 | 0.0922 | 1 | 0.1747 | 0.0582 | 0.7671 | 0.0665 | 4.358 | 0.0286 | 4.358 | 0 | 0 | —— | —— | —— | —— | —— | —— |
| ≥60 | Precision 7 | 1.0000 | 0.0154 | 0.0053 | 0.0194 | 0.0874 | 0 | 0.1731 | 0.0473 | 0.7797 | —— | —— | —— | —— | 1 | 1 | 0.3527 | 0.2751 | 0.0377 | 4.253 | 0.0093 | 4.253 |
| ≥60 | Precision 8 | 1.0000 | 0.0154 | 0.0053 | 0.0194 | 0.0874 | 0 | 0.1731 | 0.0473 | 0.7797 | —— | —— | —— | —— | 1 | 0 | 0.3527 | 0.2751 | 0.0377 | 4.253 | —— | —— |
| **Reference sources** | | **[22]** | **[22]** | **[22]** | **[22]** | **[22]** | **—** | **[16]** | **[16]** | **[16]** | **[16]** | **[16]** | **[16]** | **[16]** | **—** | **—** | **[16]** | **[16]** | **[16]** | **[16]** | **[16]** | **[16]** |
| **^a^** HIR: High Incident-risk Rate in the prediction model at baseline according to adopted cutoff value and sensitivity (“1” refers to not using prediction model and screening for all enrolled participants at baseline).  **^b^** R_SDA: Detection rate of SDA cases for ESCC in the initial examination.  **^c^** R_MoD: Detection rate of Moderate dysplasia cases in the initial examination.  **^d^** R_MD: Detection rate of Mild dysplasia cases in the initial examination.  **^e^** R_U: Detection rate of subjects with visualization of unstained areas and non-dysplasia pathology diagnosis in the initial examination.  **^f^** PS: Adopting pathology-based surveillance. “0” refers to negative and “1” refers to positive.  **^g^** P_MD: Proportion of subjects with a diagnosis of Mild Dysplasia among all subjects with a diagnosis of MD, MoD or U.  **^h^** P_MoD: Proportion of subjects with a diagnosis of Moderate Dysplasia among all subjects with a diagnosis of MD, MoD or U.  **^i^** P_U: Proportion of subjects with a diagnosis of Unstaining and non-dysplasia among all subjects with a diagnosis of MD, MoD or U.  **^j^** In_MoD: Incidence rate (per person year) of SDA cases in the surveillance among subjects with a diagnosis of Moderate Dysplasia. (“—” refers to no pathology-based surveillance adopted)  **^k^** F_MoD: Average follow-up interval (years) between baseline screening and re-examination for subjects with a diagnosis of Moderate Dysplasia.  **^l^** In_MD: Incidence rate (per person year) of SDA cases in the surveillance among subjects with a diagnosis of Mild Dysplasia. (“—” refers to no pathology-based surveillance adopted)  **^m^** F_MD: Average follow-up interval (years) between baseline screening and re-examination for subjects with a diagnosis of Mild Dysplasia.  **^n^** RS: Adopting Risk-based Surveillance. “0” refers to negative and “1” refers to positive.  **^o^** SI: Surveillance for Intermediate high-risk subjects. “0” refers to negative and “1” refers to positive. (“—” refers to no risk-based surveillance adopted)  **^p^** R_high: Rate of high progression risk. (“—” refers to no risk-based surveillance adopted)  **^q^** R_inter: Rate of intermediate-high risk. (“—” refers to no risk-based surveillance adopted)  **^r^** In_high: Incidence rate (per person year) of SDA cases in the surveillance among subjects with high progression risk. (“—” refers to no risk-based surveillance adopted)  **^s^** F_high: Average follow-up interval (years) between baseline screening and re-examination for subjects with high progression risk.  **^t^** In_inter: Incidence rate (per person year) of SDA cases in the surveillance among subjects with intermediate high progression risk. (“—” refers to no risk-based surveillance adopted)  **^u^** F_inter: Average follow-up interval (years) between baseline screening and re-examination for subjects with intermediate high progression risk. | | | | | | | | | | | | | | | | | | | | | | |
